# RETRACTED: Alper et al. Post-Traumatic Growth and Quality of Life among World Trade Center Health Registry Enrollees 16 Years after 9/11. *Int. J. Environ. Res. Public Health* 2022, *19*, 9737

**DOI:** 10.3390/ijerph22111612

**Published:** 2025-10-23

**Authors:** Howard E. Alper, Leen Feliciano, Lucie Millien, Cristina Pollari, Sean Locke

**Affiliations:** 1 World Trade Center Health Registry, New York City Department of Health and Mental Hygiene, Division of Epidemiology, New York, NY 11101, USA; 2 Graduate School of Public Health and Health Policy, The City University of New York, New York, NY 10027, USA

The Journal retracts the article “Post-Traumatic Growth and Quality of Life among World Trade Center Health Registry Enrollees 16 Years after 9/11” [1], cited above.

Following publication, the authors contacted the Editorial Office to report discovered data inaccuracies that resulted from an error in the sample frame drawn during study implementation.

Adhering to our standard procedure, an investigation was conducted by the Editorial Office and Editorial Board, which confirmed that, in order to resolve these issues, a significant number of changes in the analysis and presentation of the data is required that falls out side of the scope of a correction. As a result, the Editorial Board has decided to retract this article [1] as per MDPI’s retraction policy (https://www.mdpi.com/ethics#_bookmark30, accessed on 5 September 2025) and invite the authors to resubmit a revised version.

This retraction was approved by the Editor-in-Chief of *IJERPH*.

The authors have agreed to this retraction.
